# Supplementary material for: Specialized germline P-bodies are required to specify germ cell fate in Caenorhabditis elegans embryos
Source: Development. 2022 Nov 8;149(21):dev200920. doi: 10.1242/dev.200920 (PMC9686995; doi:10.1242/dev.200920)
Supplement: Supplementary information [file develop-149-200920-s1.pdf]

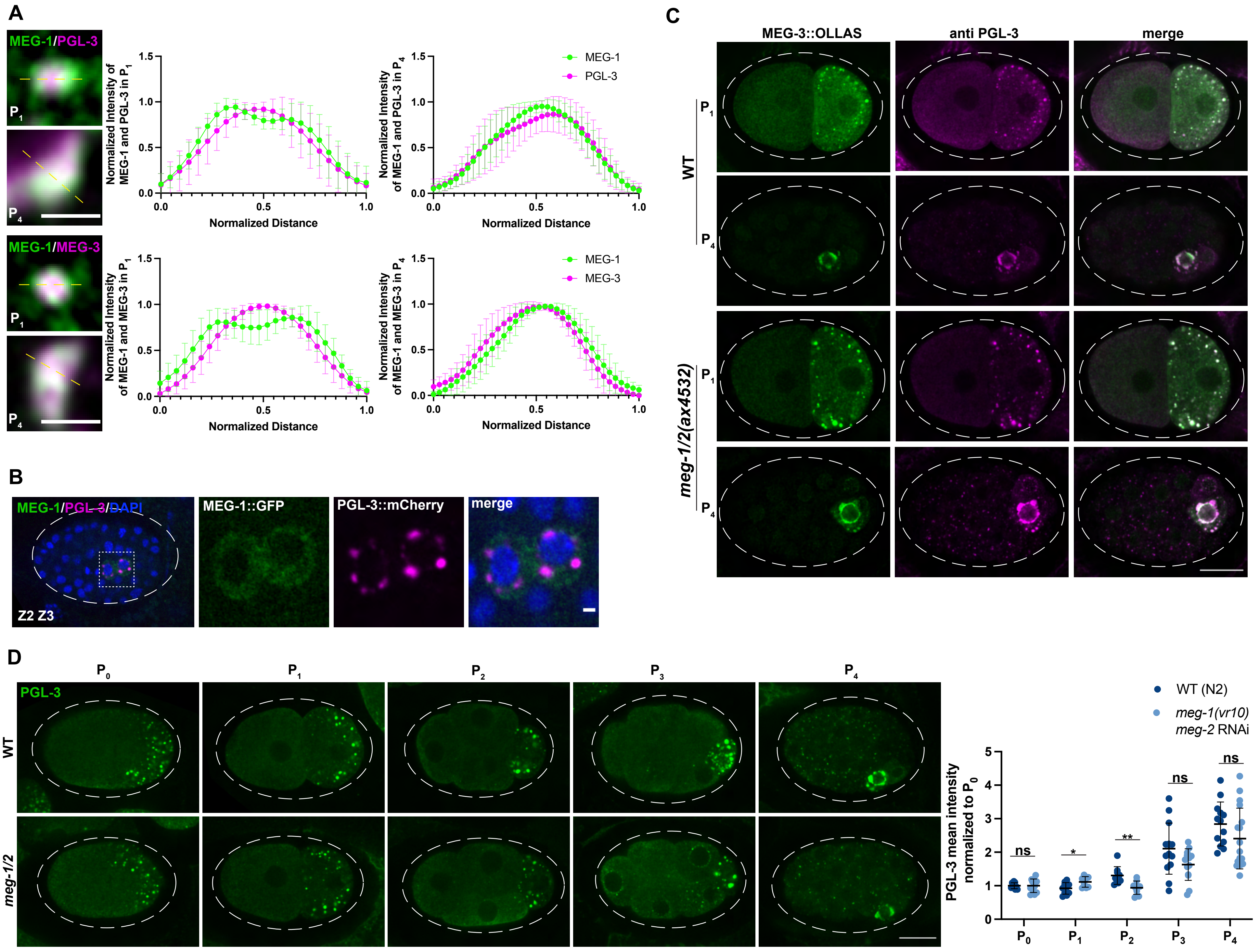

Development • Supplementary information

Fig. S1. MEG-1 puncta are distinct from P granules.
(A) Airyscan photomicrographs of granules from embryos expressing MEG-1::GFP and co-stained for GFP and PGL-3 or MEG-3::OLLAS. Dashed yellow lines indicate where intensity measurements were made for quantification. Plots show relative intensity through the center of the granules of MEG-1 and PGL-3 in P_1 (n=12 granules from 2 embryos) and P_4 (n=9 granules from 2 embryos) and MEG-1 and MEG-3 in P_1 (n=17 granules from 2 embryos) and P_4 (n=8 granules from 2 embryos). Error bars represent mean ± s.d. Scale bars are 1 µm. (B) Representative photomicrograph of a ~100-cell embryo expressing MEG-1::GFP and PGL-3::mCherry. Z2 and Z3 are shown in inset. At this stage, MEG-1 becomes dispersed in the cytoplasm, whereas PGL-3 remains in perinuclear granules. Scale bar is 1 µm. (C) Representative Airyscan photomicrographs of the indicated genotypes expressing MEG-3::OLLAS and co-stained for OLLAS and PGL-3. P granule assembly and segregation to germline blastomeres is not dependent on *meg-1 meg-2*. Scale bar is 10 µM. (D) Representative photomicrographs of embryos of the indicated genotype stained for PGL-3. In this and all subsequent figures, the designation *meg-1/2* refers to *meg-1(vr10) meg-2* RNAi. PGL-3 mean intensity in P blastomeres was normalized to P_0. On average, PGL-3 accumulation into P_2 was less efficient in *meg-1 meg-2* embryos, but was not significantly different by the P_4 stage. Scale bar is 10 µm. Data shown are from two independent experiments where mutant and control animals were processed in parallel. Number of embryos quantified per stage for WT: P_0=9, P_1= 10, P_2= 9, P_3=14, P_4=13. For *meg-1/2*: P_0=11, P_1=8, P_2=9, P_3=13, P_4=17.
***P*≤0.01, **P*≤0.05, ns=not significant (*t*-test). Error bars represent mean ± s.d.

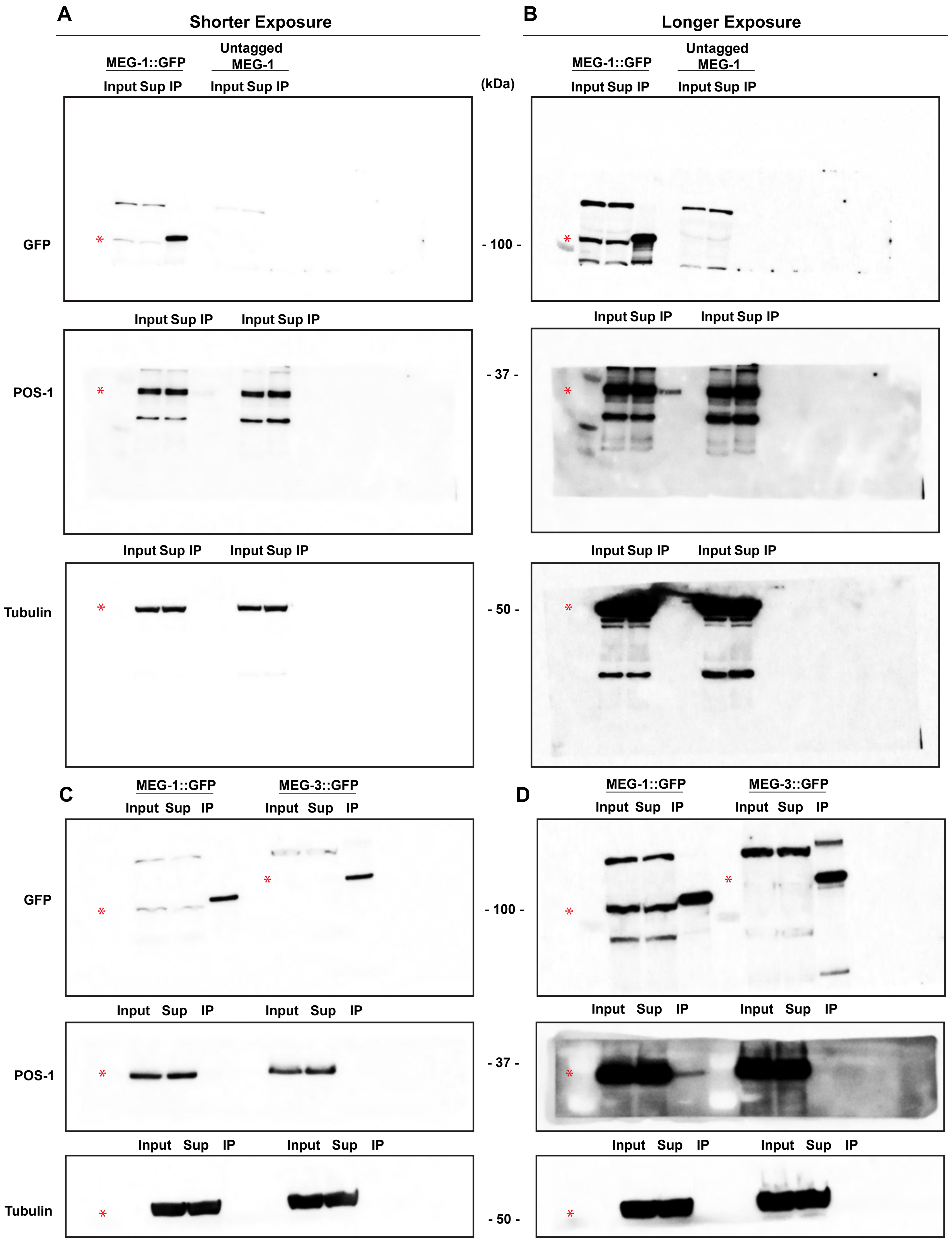

Development • Supplementary information

Fig. S2. Western blots of MEG-1::GFP immunoprecipitations.
Full western blot images from immunoprecipitation experiment shown in Fig. 3B (A and B) and 3C (C and D). Shorter exposures are shown in (A) and (C) and longer exposures of the same blot are shown in (B) and (D). Red asterisks indicate where the expected protein band would migrate. All other bands on the blots are non-specific.

Development • Supplementary information

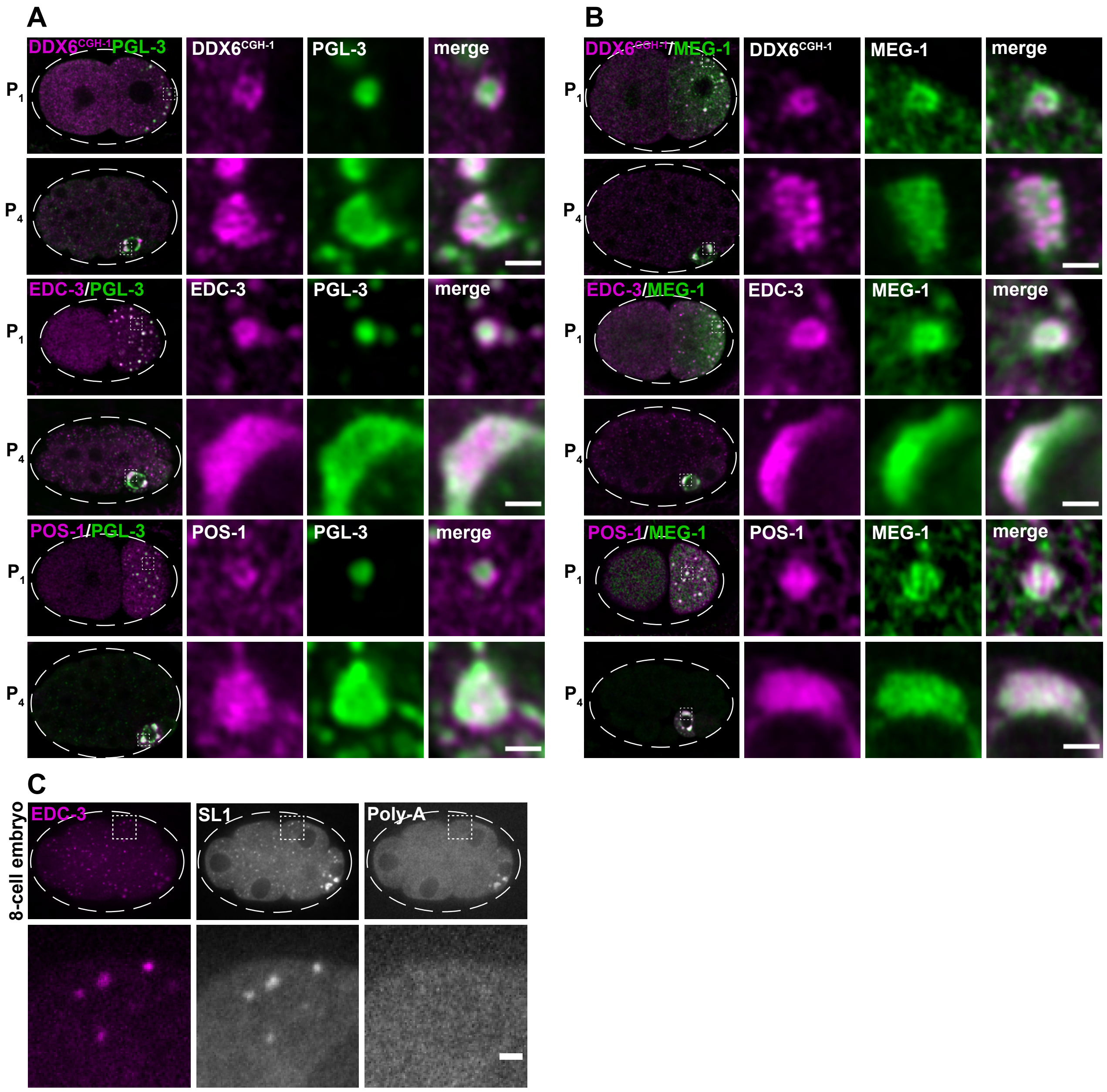

Fig. S3. Distribution of germline P-body proteins.

(A) Airyscan photomicrographs of embryos either stained for DDX6^{CGH-1}, expressing mNG::3xFLAG::EDC-3, or stained for POS-1 and co-stained for PGL-3. Insets show granules in P_1 and P_4. In P_1, P-body proteins are enriched at the periphery of PGL-3. In P_4, they overlap with PGL-3. (B) Airyscan photomicrographs of embryos expressing MEG-1::GFP and either stained for DDX6^{CGH-1}, expressing mNG::3xFLAG::EDC-3, or stained for POS-1. Insets show granules in P_1 and P_4. In P_1, MEG-1 and P-body proteins form complex partially overlapping patterns. In P_4, they overlap. (C) Represen-tative photomicrograph of 8-cell embryo expressing mNeonGreen::3xFLAG::EDC-3 and probed for SL1 and poly-A. Note that P granules (right most cell) are positive for both. Inset shows a somatic cell where EDC-3 foci are enriched for SL1, but not poly-A. Scale bars are 1 μm.

Development • Supplementary information

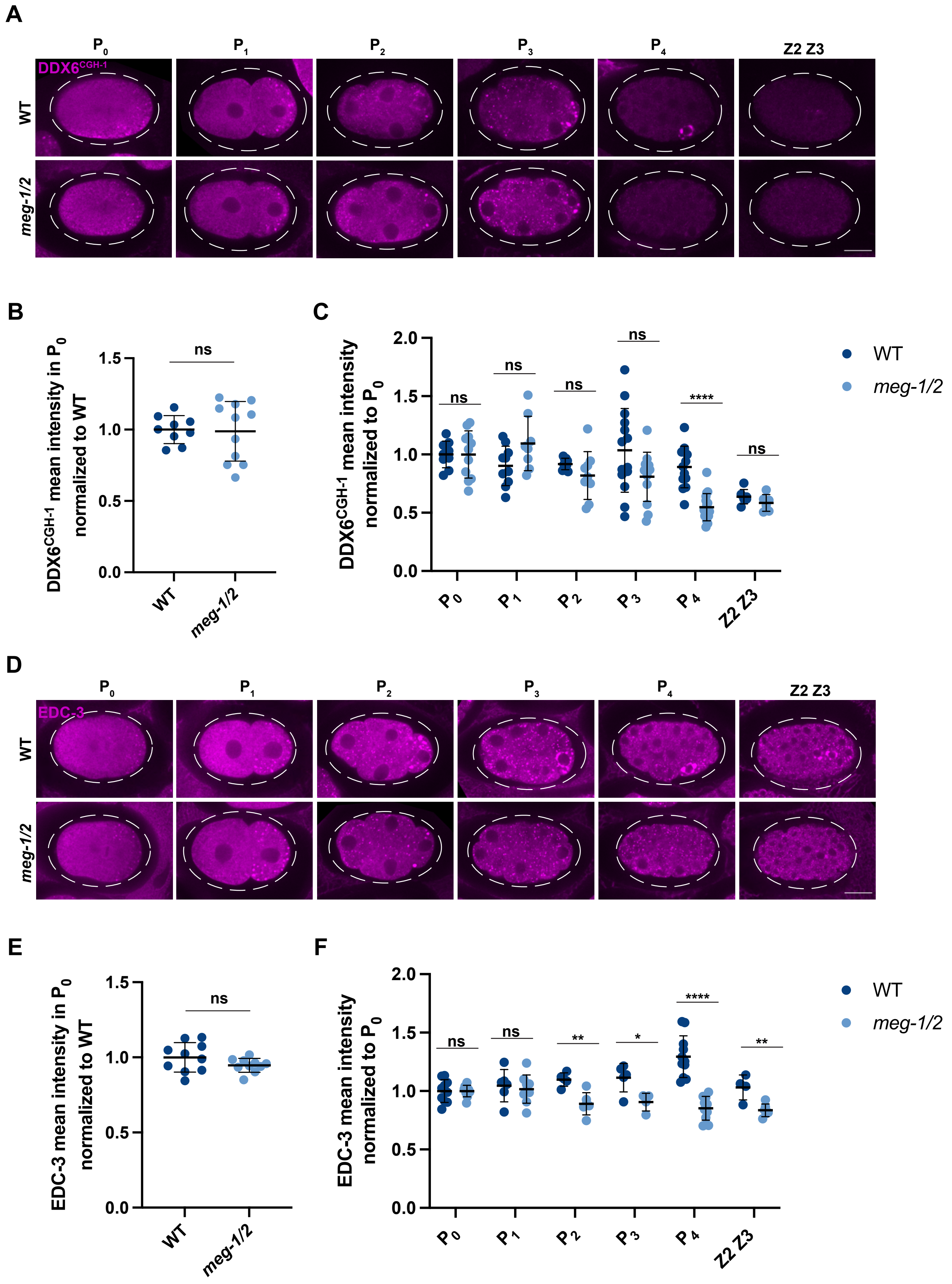
A
P0
P1
P2
P3
P4
Z2 Z3
DDX6CGH-1
WT
meg-1/2
B
DDX6CGH-1 mean intensity in P0 normalized to WT
ns
1.5
1.0
0.5
0.0
WT
meg-1/2
C
DDX6CGH-1 mean intensity normalized to P0
2.0
1.5
1.0
0.5
0.0
ns
ns
ns
ns
****
ns
P0
P1
P2
P3
P4
Z2 Z3
WT
meg-1/2
D
P0
P1
P2
P3
P4
Z2 Z3
EDC-3
WT
meg-1/2
E
EDC-3 mean intensity in P0 normalized to WT
ns
1.5
1.0
0.5
0.0
WT
meg-1/2
F
EDC-3 mean intensity normalized to P0
2.0
1.5
1.0
0.5
0.0
ns
ns
**
*
****
**
P0
P1
P2
P3
P4
Z2 Z3
WT
meg-1/2

Fig. S4. *meg-1* and *meg-2* are required to stabilize DDX6^{CGH-1} and EDC-3 in P_4.

(A) Photomicrographs of embryos of the indicated genotypes stained for DDX6^{CGH-1}. DDX6^{CGH-1} distribution is unchanged in *meg-1 meg-2* embryos compared to wild-type up to the 8-cell stage. After the 8-cell stage, DDX6^{CGH-1} is turned over in somatic blasto-meres in wild-type, and in all cells in *meg-1 meg-2* embryos. Shortly after the 100-cell stage, DDX6^{CGH-1} is uniformly present in low concentrations in both genotypes. (B) Total levels of maternally provided DDX6^{CGH-1} to P_0 zygotes are the same in *meg-1 meg-2* embryos compared to wild-type. (C) Mean intensity of DDX6^{CGH-1} in each P blastomere normalized to P_0. Quantification for each genotype is from two independent experiments where mutant and control animals were processed in parallel. Number of embryos quantified per stage for WT: P_0=9, P_1=10, P_2=9, P_3=14, P_4=13, Z2 Z3=7. For *meg-1/2*: P_0=11, P_1=8, P_2=9, P_3=13, P_4=17, Z2 Z3=6. (D) Photomicrographs of embryos of the indicated genotypes expressing mNG::3xFLAG::EDC-3 and stained for FLAG. Begin-ning in P_2, EDC-3 distribution is lower in *meg-1 meg-2* embryos compared to wild-type and is most dramatically reduced in P_4. (E) Total levels of maternally provided EDC-3 to P_0 zygotes are the same in *meg-1 meg-2* embryos compared to wild-type. (F) Mean intensity of EDC-3 in each P blastomere normalized to P_0. Quantification for each geno-type is from one experiment where mutant and control animals were processed in parallel. Number of embryos quantified per stage for WT: P_0=10, P_1=6, P_2=5, P_3=5, P_4=11, Z2 Z3=4. For *meg-1/2*: P_0=10, P_1=9, P_2=6, P_3=4, P_4=10, Z2 Z3=6. ****$P \leq 0.0001$, **$P \leq 0.01$, *$P \leq 0.05$, ns=not significant (*t*-test). All error bars represent mean ± s.d. All scale bars are 10 μm.

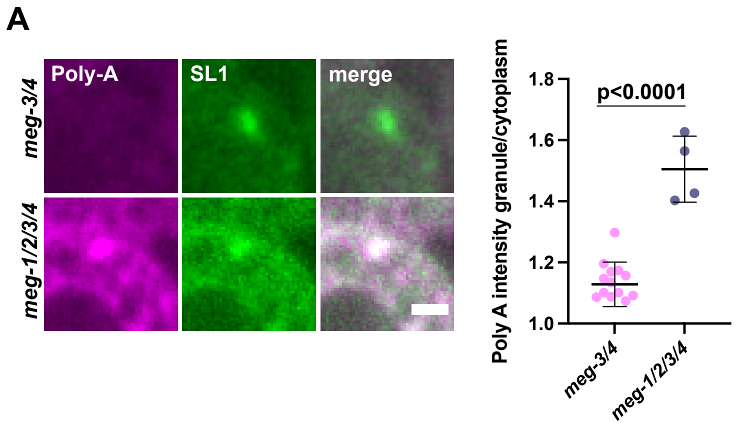

Fig. S5. Distribution of poly-A in SL1 puncta.

(A) Photomicrographs of SL1 foci in P_4 of the indicated genotypes. SL1 foci do not enrich poly-A in *meg-3 meg-4* embryos, but do in *meg-1 meg-2 meg-3 meg-4*. Embryos quantified are from the experiment shown in Fig. 4D, but only including embryos that had SL1 foci in the center z-plane of P_4. For *meg-3/4* n=14, for *meg-1/2/3/4* n=4. Scale bar is 1 µm. A *t*-test was used to make comparisons between genotypes. Error bars represent mean ± s.d.

Development • Supplementary information

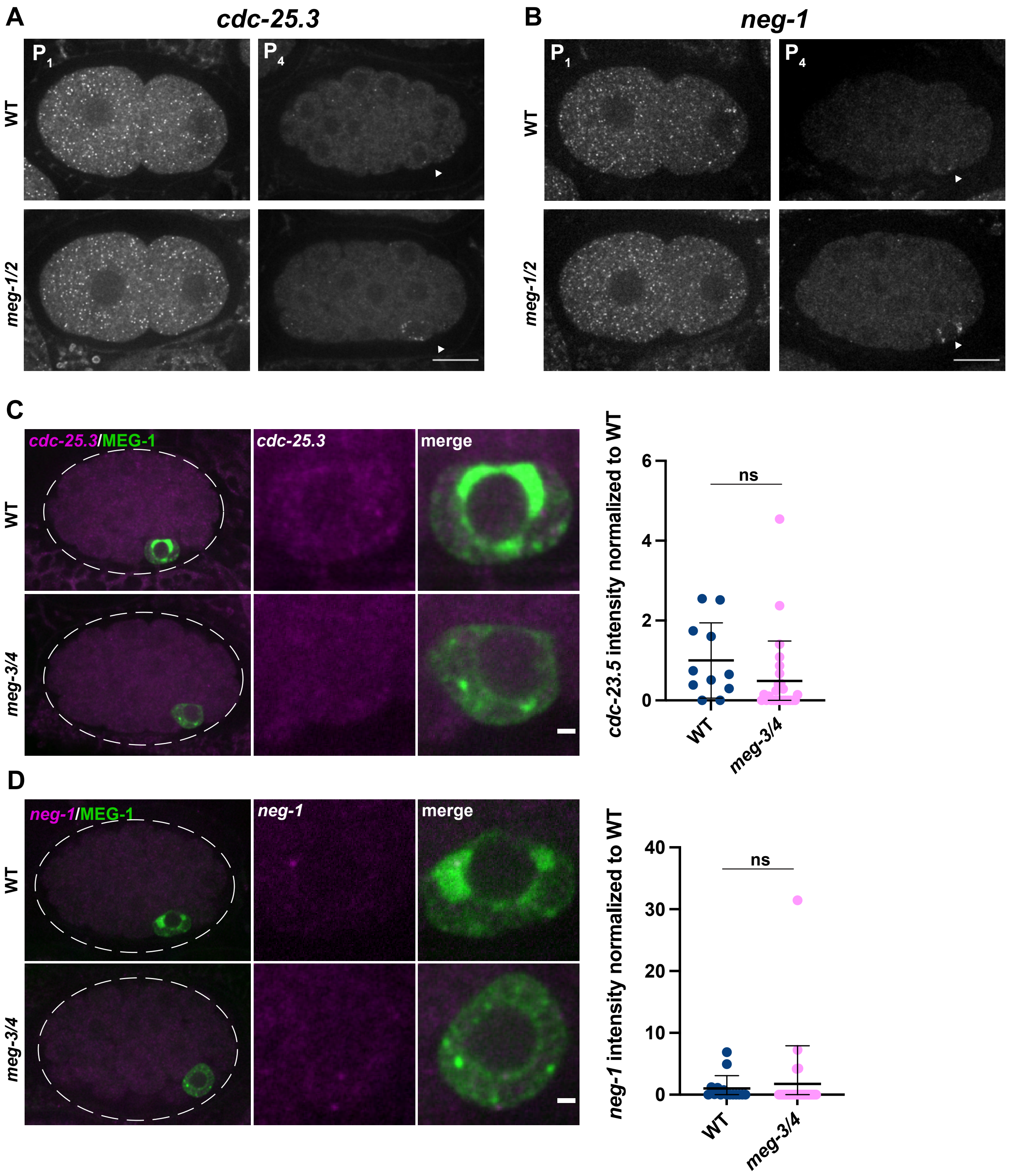

Fig. S6. Spatiotemporal localization of *cdc-25.3* and *neg-1*.

Representative photomicrographs of embryos of the indicated genotypes probed for *cdc-25.3* (A) and *neg-1* (B). Both transcripts are maternally deposited and mostly turned over by the 28-cell stage, except in P_4 in *meg-1 meg-2* embryos. Arrows point to P_4. Scale bars are 10 µm. (C) Representative photomicrographs of embryos of the indicat-ed genotypes expressing MEG-1::GFP and probed for *cdc-25.3.* Embryos were quanti-fied from two independent experiments where mutant and control animals were processed in parallel. Wild type n=11, *meg-3/4* n=26. (D) Representative photomicro-graphs of embryos of the indicated genotypes expressing MEG-1::GFP and probed for *neg-1*. Embryos were quantified from two independent experiments where mutant and control animals were processed in parallel. Wild type n=15, *meg-3/4* n=27. *cdc-25.3* and *neg-1* are efficiently turned over in *meg-3 meg-4* embryos. Scale bars in (C) and (D) are 1 µm. Error bars represent mean ± s.d. A *t*-test was used to make comparisons between genotypes.

Development • Supplementary information

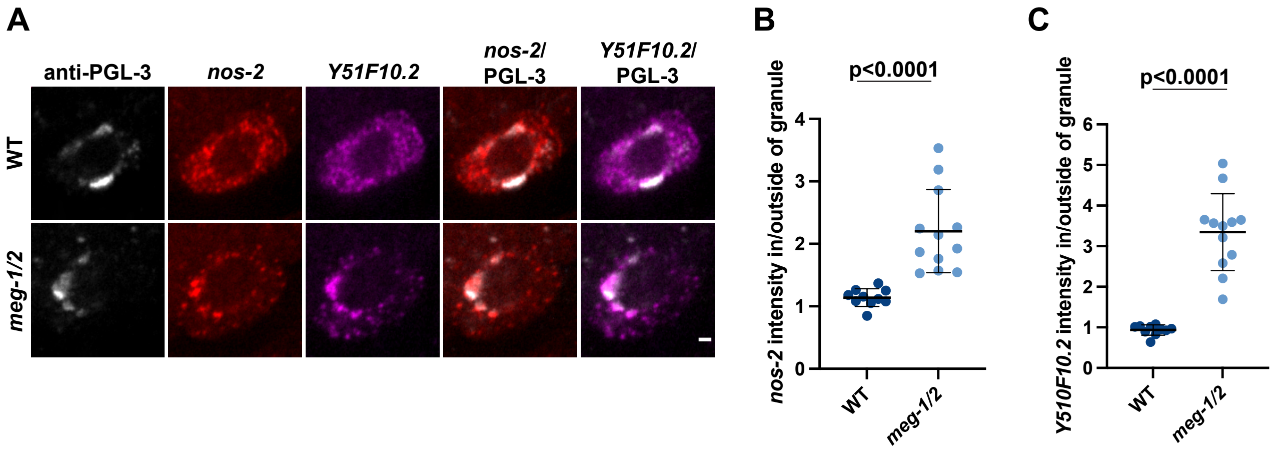

Fig. S7. *nos-2* and *Y51F10.2* RNAs are enriched in P granules in *meg-1 meg-2* P_4 blastomeres.
(A) Photomicrographs of P_4 in the indicated genotypes stained for PGL-3 and probed for *nos-2* and *Y51F10.2*. Scale bar is 1 µm. The ratio of *nos-2* (B) and *Y51F10.2* (C) inside vs. outside of the PGL-3 granule is quantified. In *meg-1 meg-2* embryos, these tran-scripts are more enriched in the granule compared to wild-type. Quantification for each genotype is from one experiment where mutant and control animals were processed in parallel. Number of embryos quantified for wild type=10; for *meg-1/2*=12. A *t*-test was used to make comparisons between genotypes. Error bars represent mean ± s.d.

Development • Supplementary information

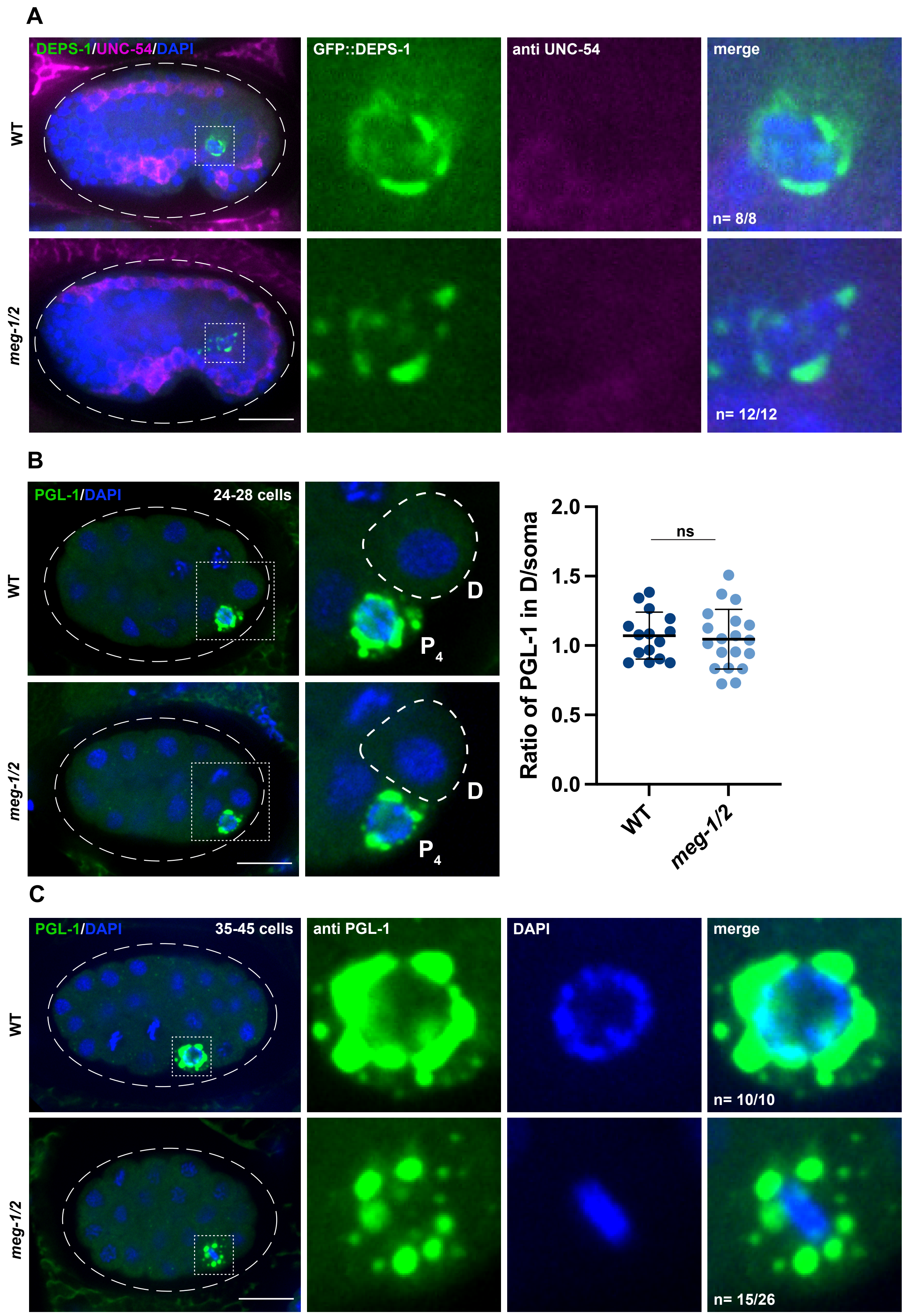

Development • Supplementary information

Fig. S8. Behavior of germ cells in *meg-1 meg-2* embryos.
(A) Photomicrographs of bean stage embryos of the indicated genotypes expressing DEPS-1::GFP and stained for myosin heavy chain (UNC-54). PGL-1 positive cells in *meg-1 meg-2* embryos do not express myosin despite transcribing *hlh-1* (Fig 7). (B) Photomicrographs of 24-28 cell stage embryos of the indicated genotypes stained for PGL-1. Inset shows P_4 and its sister cell, D. The ratio of PGL-1 intensity in D over a neighboring somatic cell was not different in *meg-1 meg-2* embryos compared to wild-type, indicating that PGL-1 is properly asymmetrically distributed to P_4 in *meg-1 meg-2* embryos. Quantification for each genotype is from one experiment where mutant and control animals were processed in parallel. Number of embryos quantified for wild type=15; for *meg-1/2*=19. A *t*-test was used to make comparisons between genotypes. Error bars represent mean ± s.d. (C) Photomicrographs of 35-45 cell stage embryos of the indicated genotypes stained for PGL-1. Inset shows P_4. 10/10 wild-type embryos had characteristic chromatin organization in P_4, whereas in 15/26 *meg-1 meg-2* embryos, P_4 began to divide precociously, indicated by chromatin in metaphase (as shown) or other stages of mitosis. All scale bars are 10 μm.

Development • Supplementary information

Table S1. Sterility of *meg-1 meg-2 (ax4532)* mutants.

Click here to download Table S1

Table S2. MEG-1 interacting proteins.

Click here to download Table S2

Table S3. Differentially expressed genes in *meg-1(vr10) meg-2* RNAi.

Click here to download Table S3

Table S4. Strains used in this study.

Click here to download Table S4
