# Supplementary material for: Specialized germline P-bodies are required to specify germ cell fate in Caenorhabditis elegans embryos
Source: Development. 2022 Nov 8;149(21):dev200920. doi: 10.1242/dev.200920 (PMC9686995; doi:10.1242/dev.200920)
Supplement: Supplementary file 1 [file develop-149-200920_review_history.pdf]

Specialized germline P bodies are required to specify germ cell fate in *C. elegans* embryos

Madeline Cassani and Geraldine Seydoux
DOI: 10.1242/dev.200920

Editor: Swathi Arur

Review timeline

Original submission:	8 May 2022
Editorial decision:	1 June 2022
First revision received:	17 August 2022
Accepted:	6 September 2022

Original submission

First decision letter

MS ID#: DEVELOP/2022/200920

MS TITLE: Specialized germline P bodies are required to specify germ cell fate in *C. elegans* embryos

AUTHORS: Madeline Cassani and Geraldine Seydoux

I have now received all the referees' reports on the above manuscript, and have reached a decision. As you will see, the referees are enthusiastic about the work, and also recommend edits that will greatly enhance the clarity of the study. All three reviewers suggest textual changes to clarify the observations, and in some of the suggestions may require additional analysis or experimentation. Some experiments to consider or to address in discussion are as suggested by reviewer 2, the claim that *meg-1* and *meg-2* drive somatic cells to germline fate. The GFP IP experiment suggested by reviewer 3 while a good control is not essential for the revision, however, I do ask that you address how you may have controlled for the non-specific interactions due to the presence of GFP. I invite you to consider all the recommendations from the reviewers' and submit a revision.Please clearly highlight all changes made in the revised manuscript and avoid using 'Tracked changes' in Word files as these are lost in PDF conversion. I should be grateful if you would also provide a point-by-point response detailing how you have dealt with the points raised by the reviewers in the 'Response to Reviewers' box. If you do not agree with any of their criticisms or suggestions please explain clearly why this is so.

Reviewer 1

Advance summary and potential significance to field

In this study, Cassani and Seydoux define the role of MEG-1 and MEG-2 in stabilizing P-body condensates that include components like POS-1 to specify germ cell founders during embryogenesis. The findings are intriguing and implicate P bodies, rather than P granules, as critical germ cell determinants, representing a significant advance toward our understanding of germ cell specification through cytoplasmic determinants. However, the following points must be addressed to strengthen and support the central hypothesis.

© 2022. Published by The Company of Biologists under the terms of the Creative Commons Attribution License (https://creativecommons.org/licenses/by/4.0/).

Comments for the author

Figure 1 – The legend states that MEG-1 granules appear smaller since they are no longer concentrated around P granules in meg-3/4 embryos. Consider adding this quantification or profile plots of MEG-1 in the meg-3/4 background to support this statement. For example, the legend statement implies that the MEG-1 profile plot in P1 no longer has the central dip in meg-3/4. Is that the case?
Figure 2 – It is unclear why MEG-1 colocalization with DDX6 and EDC-3 is shown in P4 instead of P1 where MEG-1 is at the periphery of PGL-3. Instead, P1 images are relegated to FigS4D&E where it is stated and shown that MEG-1 and DDX6 do not colocalize in P1, possibly making the case that MEG-1 puncta only correspond to germline P bodies in P4, where P granule and P body proteins overlap. If that is the case, it weakens the central conclusion of Figure 2 that MEG-1 puncta correspond to germline P bodies. Furthermore, unless I missed something, the P1 profile plots of DDX6 in Sup4A and Sup4D do not match (S4A showing peripheral DDX6 with a central profile plot dip, and S4D showing central DDX6 with no central profile plot dip). These conflicting results are further compounded by the qualifier in Sup4E that states that more complex DDX6 patterns were not included in quantifications, suggesting a degree of selection was applied prior to profiling distribution. Each of these points needs to be addressed.
Line 146. It is stated that deadenylated mRNA is accumulating in P4. I wonder if accumulating is the wrong word as it implies that SL1 (deadenylated) intensity increases from P3 to P4, which doesn't seem to be the case in 2C-D? Instead, it may be more accurate to emphasize the SL1 to PolyA ratio or that SL1 remains enriched while PolyA does not. A similar statement is made in the discussion (line 348).
Figure 3CD – It would be essential to include whether MEG-1 colocalizes with POS-1 in P1 and P4 to understand whether this is a transient association, mainly because Fig1 and S4 show PGL-3 colocalizing with MEG-1/POS-1/EDC-3 at this stage, but not in P1.
Figure 6A-D. It is unclear how RNA intensity is being measured. For example nos-2 and Y51F10.2 RNA intensity looks much higher in meg-1/2 mutants in the pictures yet is half as intense in the neighboring graph. The RNA/protein ratios change, but from the images shown, I do not see a decrease in RNA intensity in meg-1/2.
The discussion paragraph starting on line 317 is unclear and appears to contain some discrepancies. For example, line 321 references an interfacial MEG layer.
Does this include MEG-1/2/3/4? This statement is complicated because MEG-1 and MEG-3 are in different layers in P1 (Fig1F). Then a few lines later, it is stated that germline P bodies contain MEG-1/2 and MEG-3/4. This questions how germline P bodies and P granules are being defined and distinguished, and this needs to be exceptionally clear here where it is being put forth, for the first time, that germline P bodies are responsible for specifying germ cell fate. Line 333 in this paragraph states that MEG-1 enriches at the periphery of germline P body condensates. It should be clarified that this is more the case in P1 (S4D-E), as complete MEG-1 colocalization with EDC-3 and DDX6 is observed in P4 meg-3/4 animals (2A-B).
Lines 298 and 381. Depletion of several factors can cause the missegregation of P granule components into C and D lineages (mes-1, for example – Strome et al. 1995). Because of this, observing multiple P-granule positive cells in the late embryo is often the result of P granule missegregation instead of PGC proliferation. Statement of premature proliferation or that they divide precociously will first need to be validated (e.g., time-lapse imaging in embryos). For example, in mutants like lin-35 or daf-18 where premature germline proliferation is observed in L1s, it is my understanding that they still hatch with only 2 PGCs. If germ cells in mes-1/2 worms proliferate in the embryo before hatching, more imaging will be needed to support this intriguing observation.

<u>Reviewer 2</u>

Advance summary and potential significance to field

The paper by Cassani & Seydoux presents imaging, biochemical, and genetic studies that identify P bodies in the P cells of C. elegans embryos as germ-plasm bodies that are required for proper germ cell fate. Their major findings include: 1) Germline P bodies are distinct from P granules. Those granule types are adjacent in early P cells but overlap in their distributions by P4. 2) Germline P bodies contain classical P-body markers, numerous RNA-binding proteins including POS-1, and MEG-

© 2022. Published by The Company of Biologists under the terms of the Creative Commons Attribution License (https://creativecommons.org/licenses/by/4.0/).

1 and MEG-2. MEG-1 is at the periphery. 3) MEG-1 and MEG-2 are not required to assemble germline P bodies but are required to stabilize them in P4. They are not required to maintain POS-1 levels in P4. 4) By the P4 stage, the RNAs in germline P bodies retain SL1 but show reduced levels of polyA compared to earlier P cells. Conversely, meg-1 meg-2 mutant P4 cells have increased levels of poly A and increased levels of some maternal mRNAs targeted for deadenylation by POS-1. These findings reveal a role for germline P bodies in deadenylation and turn-over of some maternal mRNAs. 5) Germline P bodies also serve a role in promoting translation of some germline-critical maternal mRNAs in P4. 6) Loss of MEG-1 and MEG-2 causes P4 to aberrantly express the muscle transcription factor gene hlh-1 and to continue dividing beyond Z2/Z3 during embryogenesis, suggesting a loss of germ cell fate.

This study represents a major advance in understanding the roles of diverse granules in C. elegans embryos and how the germline is properly launched. The experiments and findings are nicely documented and discussed and justify the conclusions of the authors.

Comments for the author

My lingering question at the end of the paper was “What is the fate of P4 in meg-1 meg-2 mutants?” The presence of hlh-1 mRNA in P4 suggests transformation toward a muscle fate, as seen in pos-1 and mes-1 mutant embryos. In both of the latter, P4-derived cells are thought to become muscle (they are contractile in pos-1 mutant embryos, and they join the musculature in mes-1 mutant larvae). Given the pos-1 and mes-1 findings, I would like to see a whole-L1 image showing that in meg-1 meg-2 mutants, P4-derived cells do not join the musculature (as stated in the text), and some later larval images showing what becomes of them – do they fail to proliferate beyond the ball of cells in L1s? do they die? The claim that germline P bodies are required to specify germline fate implies that in meg-1 meg-2 mutants, Z2/Z3 do not newly express germline genes (e.g. pgl-1). Was that examined? A test for sufficiency of germline P bodies to specify germline fate would be testing the ability of ectopic MEG-1 and MEG-2 to drive a somatic cell type(s) toward germline. That is not a requested new experiment, but instead curiosity if there are any clues/predictions about that.

Other comments:
- I suggest incorporating the Fig. S1A cartoon into a figure in the main paper.
- In the Fig. 4 legend, the asterisk definitions need to be fixed.
- In swarm plots throughout the paper (e.g. Fig. 4 and Fig. S5), the median bar should be brought to the front or bolded, to be visible.
- Fig. 6A-D – In panels A & C, the levels of nos-2 and Y51F10.2 RNA look higher in meg-1/2 than in WT, but are shown as lower in the B & D swarm plots.
- Line 196 – Change CGH-1 to DDX6(superscript CGH-1) to be consistent with elsewhere.
- The appropriate citation for P4 developing as muscle in mes-1 mutant embryos is Strome et al. 1995, not Berkowitz et al. 2000.

Reviewer 3

Advance summary and potential significance to field

Cassini et al demonstrate that the disordered proteins, MEG-1 and MEG-2 contribute to P body stability in the C. elegans germline founder cell and are required for specification of germline identity. Previous work from this lab and others has shown that the related disordered proteins, MEG-3 and MEG-4, are necessary for P granule assembly and RNA recruitment to the P granules. In contrast to germ granules in other animals, they are not required for proper regulation of these RNAs or for germline specification. Therefore, the question of how specification of the germline is regulated in C. elegans has remained unanswered. Previous confocal imaging studies showed that a second class of granules, P bodies, associate with the surface of P granules. In this study, the authors use super resolution imaging and IP/MS to show that MEG1/2, previously described as P granule proteins, more closely associate with P body components on the surface of P granules. The remainder of the paper analyzes meg1/2 and meg1/2/3/4 deletion mutants to characterize the role of MEG1/2. The authors provide some evidence that MEG1/2 are necessary for proper maintenance of the P bodies, although this argument should be strengthened with further experiments

© 2022. Published by The Company of Biologists under the terms of the Creative Commons Attribution License (https://creativecommons.org/licenses/by/4.0/).

(see #1 below). Using RNA seq, smFISH and immunofluorescence, the authors show that RNAs, including targets of POS-1, are widely mis-regulated in the absence of MEG1/2, including aberrant stability of somatic determinants and a failure to translate germline determinants. Specification of the germline is also disrupted in these mutants.

Overall, this is an interesting paper that supports a role for MEG-1/2 in post-transcriptional regulation in the P4 cell necessary for germline specification.
The authors propose that this necessity is due to the role of MEG-1/2 in P body maintenance. However, another role of MEG-1/2 cannot be ruled out and additional evidence to support the role of P bodies as critical drivers of germline fate specification would greatly strengthen the manuscript.

Comments for the author

Major points to address
1)The authors should check for colocalization of various P body components at P4 and determine if any of these factors overlap with the POS-1 puncta in meg1/2 mutants. This will allow the authors to determine if there are any intact P bodies. Furthermore, the authors claim that P body assembly is not affected in meg1/2 mutants, but only show DDX6 distribution at earlier embryonic stages. Other P body proteins and their colocalization with one another should be analyzed at these earlier stages as well.
2)In Figure 2A-B and Figure 3C, colocalization of MEG-1 with P body components is shown only in the P4 cell. Based on the results in Figure 1C-F, this appears to be a time when separation between different granule types is lost in the wildtype. To determine if the overlap is independent of this restructuring, this analysis should be performed at earlier embryonic stages as well.
3)The authors make the claim that MEG1/2 are P body components, but in wildtype embryos, MEG-1 enriches at the periphery of DDX6 (Fig. S4) . Similar separation of DDX6 from PGL-3, another P granule protein, (Fig. 1C-F) is used to argue that these proteins are in separate granules. If the authors want to claim that MEG-1/2 are in P bodies, they must better define the criteria used to determine if two factors are in the same granule.
4)The use of untagged MEG-1 as a control for their MEG-1::GFP IP experiments does not control for the possibility of nonspecific binding of proteins to GFP. An IP with a GFP control should be included.
5)The RNAs shown to be stabilized in Fig. 5 do not localize to P granules, but appear to be concentrating in distinct puncta. What are these puncta? The authors should test if they overlap with any of the components of P bodies or of other granule types in the P4 cell.
6)Are P granule-localized RNAs stabilized in meg1/2 mutants? This should be tested.
7)As mentioned in the discussion, NOS-2 has been previously implicated in RNA turnover. Thus, the stabilization of RNAs shown in Fig. 5 could be the indirect consequence of a failure to translate NOS-2. The authors should address this possibility experimentally if possible, but if not then this caveat must be acknowledged.

Minor issues
1)The authors use both super-resolution microscopy and spinning disk confocal microscopy, but which method is used for each figure is only stated in the methods. This information should be added to the figure legends and/or the results section as well.
2)The profile plots in Figures 1-3 would be easier to interpret if an example indicating where on the image the data are acquired is included.
3)The images in Fig. 1A-B appear to show a decrease in MEG-1 levels in the P cells in meg3/4 mutants. This should be addressed by the authors.
4)The authors state that Fig. 1B shows “that MEG-1 still enriched in germplasm” in meg3/4 mutants. The authors should clarify if and how they are distinguishing germ plasm from the rest of the cytoplasm in the P cells.
5)The meg1/2 genotype shown in images shown in Fig. S1C is different from the meg1/2 genotype that quantified in S1D - the same genotype should be used for both.
6)The authors state that “P granule enrichment in P blastomeres was not as robust in meg-1 meg-2 embryos”. This statement is not supported by the data shown in Fig.
S1C-D, which indicate there is only a decrease in P granules in the P2 cell, with the same or higher levels at all other stages analyzed.This statement isn’t needed to support the main arguments, so it could be removed.

© 2022. Published by The Company of Biologists under the terms of the Creative Commons Attribution License (https://creativecommons.org/licenses/by/4.0/).

7)The legend in Fig. 3A show 507 “Not significant hits in MEG-1 IP”, but this is never described in the text. The data in this figure would be easier to interpret if both categories of hits were explained.
8)The authors say that “early embryo lysates” were used for the IP experiments.
The specific embryonic stages included in this analysis should be stated.
9)In Supplemental Figure 4D, the DDX6 distribution looks different from all the other examples in this figure. Another image with more representative DDX6 distribution should be used.
10)In Fig. 4D, the intensity of Poly A is shown to increase in meg1/2 and meg1/2/3/4 mutants, as is stated in the text, but the levels appear lower in the images shown in Fig. 4C. There is a similar issue in Fig. 6, where the quantification indicates RNA levels are decreasing, but the intensity appears brighter in the images. This should either be addressed in the text or different representative images should be chosen.
11)In the introduction, the authors discuss “P body-like condensates”, but later call these structures P bodies. It is unclear why separate terminology is being used here.
12)The authors should address the functional significance of the docking of P bodies onto the P granules in the discussion.
13)In the discussion, the authors state “In Drosophila, the DDC6/4-ET like complex (ME31B/Cup) is targeted for degradation in somatic cells”. The Drosophila embryo has not yet cellularized at the time of these events, so “somatic region” should be used instead.

First revision

Author response to reviewers' comments

We thank the reviewers for their thoughtful and constructive comments which have helped us improve our manuscript. Note that, in the revised manuscript, we have highlighted in red the text that refers to the new data we generated in response to the reviewers comments.

Reviewer 1 Advance Summary and Potential Significance to Field:
In this study, Cassani and Seydoux define the role of MEG-1 and MEG-2 in stabilizing P-body condensates that include components like POS-1 to specify germ cell founders during embryogenesis. The findings are intriguing and implicate P bodies, rather than P granules, as critical germ cell determinants, representing a significant advance toward our understanding of germ cell specification through cytoplasmic determinants. However, the following points must be addressed to strengthen and support the central hypothesis.

Reviewer 1 Comments for the Author:

Figure 1 – The legend states that MEG-1 granules appear smaller since they are no longer concentrated around P granules in meg-3/4 embryos. Consider adding this quantification or profile plots of MEG-1 in the meg-3/4 background to support this statement. For example, the legend statement implies that the MEG-1 profile plot in P1 no longer has the central dip in meg-3/4. Is that the case?

We have removed these claims from the legend since not essential for the main point of this figure.

Figure 2 – It is unclear why MEG-1 colocalization with DDX6 and EDC-3 is shown in P4 instead of P1 where MEG-1 is at the periphery of PGL-3. Instead, P1 images are relegated to FigS4D&E where it is stated and shown that MEG-1 and DDX6 do not colocalize in P1, possibly making the case that MEG-1 puncta only correspond to germline P bodies in P4, where P granule and P body proteins overlap. If that is the case, it weakens the central conclusion of Figure 2 that MEG-1 puncta correspond to germline P bodies.

The original presentation of the data was confusing and therefore we have reorganized the text and figures. We moved the Mass Spec data to Fig. 2 and gathered the characterization of P body markers and POS-1 into two figures: Fig. 3 and Fig. S3.

© 2022. Published by The Company of Biologists under the terms of the Creative Commons Attribution License (https://creativecommons.org/licenses/by/4.0/).

Fig. 3: Distribution of MEG-1 and P body markers in P4 of *meg-3 meg-4* embryos. DDX6, EDC-3 and MEG-1 coalesce in spherical puncta that are positive for SL1 and negative for poly-A, as expected for P bodies. (We clarify throughout the text that the appellation “germline P bodies” refers SPECIFICALLY to the MEG-1/EDC3/DDX6/POS- 1/SL1+polyA- puncta we observe in P4.)

Fig. S3: Distribution of MEG-1 and P body markers in P1 and P4 of wild-type embryos, to show the changing overlap.

Furthermore, unless I missed something, the P1 profile plots of DDX6 in Sup4A and Sup4D do not match (S4A showing peripheral DDX6 with a central profile plot dip, and S4D showing central DDX6 with no central profile plot dip). These conflicting results are further compounded by the qualifier in Sup4E that states that more complex DDX6 patterns were not included in quantifications, suggesting a degree of selection was applied prior to profiling distribution. Each of these points needs to be addressed.

Thank you. As stated above, we have reorganized the figures and the text to clarify all these points.

Line 146. It is stated that deadenylated mRNA is accumulating in P4. I wonder if accumulating is the wrong word as it implies that SL1 (deadenylated) intensity increases from P3 to P4, which doesn’t seem to be the case in 2C-D? Instead, it may be more accurate to emphasize the SL1 to PolyA ratio or that SL1 remains enriched while PolyA does not. A similar statement is made in the discussion (line 348).

Agreed! We have modified the text as suggested.

Figure 3CD – It would be essential to include whether MEG-1 colocalizes with POS-1 in P1 and P4 to understand whether this is a transient association, mainly because Fig1 and S4 show PGL-3 colocalizing with MEG-1/POS-1/EDC-3 at this stage, but not in P1.

We now include the POS-1 data, alongside MEG-1 and P body markers, in Fig. 3 and Fig. S3. As mentioned above, we have reorganized these data to make it clear that colocalization is seen in P4, and more complex patterns are seen earlier.

Figure 6A-D. It is unclear how RNA intensity is being measured. For example, nos-2 and Y51F10.2 RNA intensity looks much higher in meg-1/2 mutants in the pictures yet is half as intense in the neighboring graph. The RNA/protein ratios change, but from the images shown, I do not see a decrease in RNA intensity in meg-1/2.

We have replaced those panels with more representative micrographs. RNA levels appear higher in *meg-1/2* due to the RNA concentrating in P granules. The quantification, however, was based on total RNA levels throughout the cell.

The discussion paragraph starting on line 317 is unclear and appears to contain some discrepancies. For example, line 321 references an interfacial MEG layer. Does this include MEG-1/2/3/4? This statement is complicated because MEG-1 and MEG-3 are in different layers in P1 (Fig1F).

Then a few lines later, it is stated that germline P bodies contain MEG-1/2 and MEG-3/4. This questions how germline P bodies and P granules are being defined and distinguished, and this needs to be exceptionally clear here where it is being put forth, for the first time, that germline P bodies are responsible for specifying germ cell fate.

We have rewritten the discussion to clarify what we mean by these terms.

Line 333 in this paragraph states that MEG-1 enriches at the periphery of germline P body condensates. It should be clarified that this is more the case in P1 (S4D-E), as complete MEG-1 colocalization with EDC-3 and DDX6 is observed in P4 meg- 3/4 animals (2A-B).

© 2022. Published by The Company of Biologists under the terms of the Creative Commons Attribution License (https://creativecommons.org/licenses/by/4.0/).

We have removed this statement and clarified that the mechanism by which MEG-1 stabilizes DDX6 and EDC3 remains unclear.

Lines 298 and 381. Depletion of several factors can cause the missegregation of P granule components into C and D lineages (mes-1, for example – Strome et al. 1995). Because of this, observing multiple P-granule positive cells in the late embryo is often the result of P granule missegregation instead of PGC proliferation. Statement of premature proliferation or that they divide precociously will first need to be validated (e.g., time-lapse imaging in embryos). For example, in mutants like lin-35 or daf-18 where premature germline proliferation is observed in L1s, it is my understanding that they still hatch with only 2 PGCs. If germ cells in mes-1/2 worms proliferate in the embryo before hatching, more imaging will be needed to support this intriguing observation.

Excellent point. We have analyzed the pattern of P granules in 28-cell stage *meg-1 meg-2* embryos and confirmed that there are no PGL granules miss-segregated to the D blastomere. Additionally, P4 divides precociously in at least half of *meg-1 meg-2* embryos. These new findings are shown in Figure S8A-B.

Reviewer 2 Advance Summary and Potential Significance to Field:
The paper by Cassani & Seydoux presents imaging, biochemical, and genetic studies that identify P bodies in the P cells of C. elegans embryos as germ-plasm bodies that are required for proper germ cell fate. Their major findings include: 1) Germline P bodies are distinct from P granules. Those granule types are adjacent in early P cells but overlap in their distributions by P4. 2) Germline P bodies contain classical P-body markers, numerous RNA-binding proteins including POS-1, and MEG-1 and MEG-2.
MEG-1 is at the periphery. 3) MEG-1 and MEG-2 are not required to assemble germline P bodies but are required to stabilize them in P4. They are not required to maintain POS-1 levels in P4. 4) By the P4 stage, the RNAs in germline P bodies retain SL1 but show reduced levels of polyA compared to earlier P cells. Conversely, meg-1 meg-2 mutant P4 cells have increased levels of poly A and increased levels of some maternal mRNAs targeted for deadenylation by POS-1. These findings reveal a role for germline P bodies in deadenylation and turn-over of some maternal mRNAs. 5) Germline P bodies also serve a role in promoting translation of some germline-critical maternal mRNAs in P4. 6) Loss of MEG-1 and MEG-2 causes P4 to aberrantly express the muscle transcription factor gene hlh-1 and to continue dividing beyond Z2/Z3 during embryogenesis, suggesting a loss of germ cell fate.

This study represents a major advance in understanding the roles of diverse granules in C. elegans embryos and how the germline is properly launched. The experiments and findings are nicely documented and discussed and justify the conclusions of the authors.

Reviewer 2 Comments for the Author:
My lingering question at the end of the paper was "What is the fate of P4 in meg-1 meg-2 mutants?" The presence of hlh-1 mRNA in P4 suggests transformation toward a muscle fate, as seen in pos-1 and mes-1 mutant embryos. In both of the latter, P4- derived cells are thought to become muscle (they are contractile in pos-1 mutant embryos, and they join the musculature in mes-1 mutant larvae). Given the pos-1 and mes-1 findings, I would like to see a whole-L1 image showing that in meg-1 meg-2 mutants, P4-derived cells do not join the musculature (as stated in the text), and some later larval images showing what becomes of them – do they fail to proliferate beyond the ball of cells in L1s? do they die?

Excellent points! We have added new experiments to explore the fate of P4 descendants in *meg-1 meg-2* embryos. We now show that P4 descendants in *meg-1 meg-2* embryos do not express UNC-54 (myosin heavy chain). These data are shown in Fig. S8C.

The claim that germline P bodies are required to specify germline fate implies that in meg-1 meg-2 mutants, Z2/Z3 do not newly express germline genes (e.g. pgl-1). Was that examined?

© 2022. Published by The Company of Biologists under the terms of the Creative Commons Attribution License (https://creativecommons.org/licenses/by/4.0/).

We have now examined the expression of *xnd-1*, which is transcribed at the ~300 cell stage in addition to being a maternal transcript (Mainpal et al., 2015). We observe that zygotic *xnd-1* is not expressed in the majority of *meg-1 meg-2* embryos examined at this stage.

A test for sufficiency of germline P bodies to specify germline fate would be testing the ability of ectopic MEG-1 and MEG-2 to drive a somatic cell type(s) toward germline.
That is not a requested new experiment, but instead curiosity if there are any clues/predictions about that.

This is an interesting question. We added the following to the last section of the Discussion (*Limitations of this study*):
We also do not address whether MEG-1/2 or germline P bodies are merely required (permissive) or are sufficient (instructive) to specify germ cell fate. MEG-1/2 enrich preferentially into P blastomeres from the zygote-stage onward; mutations that prevent this localization may help determine whether MEG-1/2 play a permissive or instructive role in germ cell fate specification.

Other comments:
- I suggest incorporating the Fig. S1A cartoon into a figure in the main paper.

We have added the cartoon to Figure 1.

- In the Fig. 4 legend, the asterisk definitions need to be fixed.

The asterisk definitions were the actual P values for those experiments. We have changed them to generic definitions to avoid confusion.

- In swarm plots throughout the paper (e.g. Fig. 4 and Fig. S5), the median bar should be brought to the front or bolded, to be visible.

Standard deviation and mean bars have been brought to the front on all plots.

- Fig. 6A-D – In panels A & C, the levels of nos-2 and Y51F10.2 RNA look higher in meg-1/2 than in WT, but are shown as lower in the B & D swarm plots.

(See Above)
We have replaced those panels with more representative micrographs. RNA levels appear higher in *meg-1/2* due to the RNA concentrating in P granules. The quantification, however, was based on total RNA levels throughout the cell.

- Line 196 – Change CGH-1 to DDX6(superscript CGH-1) to be consistent with elsewhere.

We have corrected this - thank you

- The appropriate citation for P4 developing as muscle in mes-1 mutant embryos is Strome et al. 1995, not Berkowitz et al. 2000.

We have corrected this - thank you

Reviewer 3 Advance Summary and Potential Significance to Field: Cassini et al demonstrate that the disordered proteins, MEG-1 and MEG-2, contribute to P body stability in the C. elegans germline founder cell and are required for specification of germline identity. Previous work from this lab and others has shown that the related disordered proteins, MEG-3 and MEG-4, are necessary for P granule assembly and RNA recruitment to the P granules. In contrast to germ granules in other animals, they are not required for proper regulation of these RNAs or for germline specification. Therefore, the question of how specification of the germline is regulated in C. elegans has remained unanswered. Previous confocal imaging studies showed that a second class of granules, P bodies, associate with the surface of P granules. In this study, the authors use super resolution

© 2022. Published by The Company of Biologists under the terms of the Creative Commons Attribution License (https://creativecommons.org/licenses/by/4.0/).

imaging and IP/MS to show that MEG1/2, previously described as P granule proteins, more closely associate with P body components on the surface of P granules. The remainder of the paper analyzes meg1/2 and meg1/2/3/4 deletion mutants to characterize the role of MEG1/2. The authors provide some evidence that MEG1/2 are necessary for proper maintenance of the P bodies, although this argument should be strengthened with further experiments (see #1 below). Using RNA seq, smFISH and immunofluorescence, the authors show that RNAs, including targets of POS-1, are widely mis-regulated in the absence of MEG1/2, including aberrant stability of somatic determinants and a failure to translate germline determinants. Specification of the germline is also disrupted in these mutants.

Overall, this is an interesting paper that supports a role for MEG-1/2 in post- transcriptional regulation in the P4 cell necessary for germline specification.
The authors propose that this necessity is due to the role of MEG-1/2 in P body maintenance. However, another role of MEG-1/2 cannot be ruled out and additional evidence to support the role of P bodies as critical drivers of germline fate specification would greatly strengthen the manuscript.

Reviewer 3 Comments for the Author:

Major points to address

1) The authors should check for colocalization of various P body components at P4 and determine if any of these factors overlap with the POS-1 puncta in meg1/2 mutants. This will allow the authors to determine if there are any intact P bodies.

We have not done these experiments systematically since 1) EDC-3, CGH-1 levels are reduced in *meg-1/2* mutants and 2) the few remaining RNA-rich puncta in *meg-1/2/3/4* are polyA+, unlike "true" P bodies (Fig. 4 and text).

Furthermore, the authors claim that P body assembly is not affected in meg1/2 mutants, but only show DDX6 distribution at earlier embryonic stages. Other P body proteins and their colocalization with one another should be analyzed at these earlier stages as well.

We include new data and text describing and quantifying the distribution of both DDX6 and EDC-3 from the P0 to P4 stage (Fig. S4). These data support the hypothesis that *meg-1/2* are required primarily to maintain high levels of these proteins in P4. We also clarify in the discussion that the mechanisms by which MEG-1/2 achieve this remain unclear.

2) In Figure 2A-B and Figure 3C, colocalization of MEG-1 with P body components is shown only in the P4 cell. Based on the results in Figure 1C-F, this appears to be a time when separation between different granule types is lost in the wildtype. To determine if the overlap is independent of this restructuring, this analysis should be performed at earlier embryonic stages as well.

We have reorganized the figures to clarify the relative distribution of MEG-1 and P body proteins throughout embryogenesis (now all shown in Fig. 3 and Sup. Fig.3). MEG-1 partially overlaps with P body components in P1 and fully overlaps in P4 coincident with when MEG-1 puncta becoming SL1+polyA-, consistent with "mature P bodies".

We have clarified in the text that "germline P bodies" refer to the MEG-1 puncta observed in P4.

3) The authors make the claim that MEG1/2 are P body components, but in wildtype embryos, MEG-1 enriches at the periphery of DDX6 (Fig. S4). Similar separation of DDX6 from PGL-3, another P granule protein, (Fig. 1C-F) is used to argue that these proteins are in

© 2022. Published by The Company of Biologists under the terms of the Creative Commons Attribution License (https://creativecommons.org/licenses/by/4.0/).

separate granules. If the authors want to claim that MEG-1/2 are in P bodies, they must better define the criteria used to determine if two factors are in the same granule.

Agreed. We clarify that P body components form complex partially-overlapping assemblies around P granules in P1 and come together into well-defined puncta (best visualized in *meg-3/4* embryos) only in P4. We clarify now throughout the text that the designation "germline P bodies" refers to the P4 puncta ONLY. These foci contain MEG- 1, DDX6, EDC-3, POS-1 and SL1+polyA-.

4) The use of untagged MEG-1 as a control for their MEG-1::GFP IP experiments does not control for the possibility of nonspecific binding of proteins to GFP. An IP with a GFP control should be included.

Agreed. We now include a new experiment where we show that immunoprecipitation of MEG-3::GFP does NOT pull down POS-1. This is shown in Fig. 2C.

5) The RNAs shown to be stabilized in Fig. 5 do not localize to P granules, but appear to be concentrating in distinct puncta. What are these puncta? The authors should test if they overlap with any of the components of P bodies or of other granule types in the P4 cell.

The experiment was originally done with GFP::WAGO-4 as a germ cell marker, but WAGO-4 is distinct from P granules in P4 (Wan et al., 2018). We have repeated these experiments using a P granule marker and confirm that the stabilized RNAs accumulate in the perinuclear P granules. (Fig. 5C, 5E).

6) Are P granule-localized RNAs stabilized in meg1/2 mutants? This should be tested.

The *nos-1*, *Y51F10.2* and *xnd-1* mRNAs actually are present at LOWER levels in *meg-1 meg-2* mutant P4 compared to wild-type (Fig. 6). This difference likely is due to the fact that P granules are not as robustly enriched in P blastomeres in these mutants, or alternatively these mRNAs might be degraded as a consequence of less efficient translation.

7) As mentioned in the discussion, NOS-2 has been previously implicated in RNA turnover. Thus, the stabilization of RNAs shown in Fig. 5 could be the indirect consequence of a failure to translate NOS-2. The authors should address this possibility experimentally if possible, but if not then this caveat must be acknowledged.

We did not see stabilization of these RNAs in *meg-3 meg-4* mutants (See new data in Fig. S6C, S6D); which also have low levels of NOS-2 protein (shown in Fig. 6A, 6B). Note that although these RNAs accumulate in P granules in P4 of *meg-1/2* mutants, they are not normally present in P granules and would not be expected to be affected by loss of *meg-3/4*.

Minor issues

1) The authors use both super-resolution microscopy and spinning disk confocal microscopy, but which method is used for each figure is only stated in the methods. This information should be added to the figure legends and/or the results section as well.

We have now indicated when Airyscan is used in the figure legends.

2) The profile plots in Figures 1-3 would be easier to interpret if an example indicating where on the image the data are acquired is included.

An example and explanation has been added to profile plots in Fig. S1A.

3) The images in Fig. 1A-B appear to show a decrease in MEG-1 levels in the P cells in meg3/4 mutants. This should be addressed by the authors.

We have not quantified this difference since the main point of this figure was to confirm that MEG-1 can form puncta and segregate with germ plasm independent of P granules as already

© 2022. Published by The Company of Biologists under the terms of the Creative Commons Attribution License (https://creativecommons.org/licenses/by/4.0/).

documented by Leacock and Reinke; 2008.

4) The authors state that Fig. 1B shows “that MEG-1 still enriched in germplasm” in meg3/4 mutants. The authors should clarify if and how they are distinguishing germ plasm from the rest of the cytoplasm in the P cells.

We have replaced this phrasing with “enrich preferentially in P blastomeres”.

5) The meg1/2 genotype shown in images shown in Fig. S1C is different from the meg1/2 genotype that quantified in S1D - the same genotype should be used for both.

We thought it would be useful to confirm (Fig. S1C) that P granules still form in the new deletion mutant we created which removes both *meg-1* and *meg-2* open reading frames. Quantification was done in the *meg-1(vr10) meg-2(RNAi)* because it is easier to obtain multiple embryos from these mothers and we are now including representative pictures of that genotype used for that quantification. (Fig. S1D).

6) The authors state that “P granule enrichment in P blastomeres was not as robust in meg-1 meg-2 embryos”. This statement is not supported by the data shown in Fig. S1C-D, which indicate there is only a decrease in P granules in the P2 cell, with the same or higher levels at all other stages analyzed. This statement isn’t needed to support the main arguments, so it could be removed.

We believe the statement “not as robust” reflect the data accurately since there was a significant difference in the P2 stage.

7) The legend in Fig. 3A show 507 “Not significant hits in MEG-1 IP”, but this is never described in the text. The data in this figure would be easier to interpret if both categories of hits were explained.

The 507 hits referred to all proteins identified in MEG-1 IPs that were not significantly enriched. We have removed this from the figure to avoid confusion.

8) The authors say that “early embryo lysates” were used for the IP experiments. The specific embryonic stages included in this analysis should be stated.

We have added this information in the Methods. The embryos were obtained from bleaching of gravid adults.

9) In Supplemental Figure 4D, the DDX6 distribution looks different from all the other examples in this figure. Another image with more representative DDX6 distribution should be used.

We have rearranged these figures and provided a clearer description in the text.

10) In Fig. 4D, the intensity of Poly A is shown to increase in meg1/2 and meg1/2/3/4 mutants, as is stated in the text, but the levels appear lower in the images shown in Fig. 4C. There is a similar issue in Fig. 6, where the quantification indicates RNA levels are decreasing, but the intensity appears brighter in the images. This should either be addressed in the text or different representative images should be chosen.

Note that the levels referred to are “total cytoplasmic levels” not only the level in puncta. We have provided more representative images.

11) In the introduction, the authors discuss “P body-like condensates”, but later call these structures P bodies. It is unclear why separate terminology is being used here.

We use the term P body-like in the beginning when we are still building our hypothesis, and introduce the term “germline P bodies” later in the text after we present data to support this nomenclature.

© 2022. Published by The Company of Biologists under the terms of the Creative Commons Attribution License (https://creativecommons.org/licenses/by/4.0/).

12) The authors should address the functional significance of the docking of P bodies onto the P granules in the discussion.

We now address this point – line 381.

13) In the discussion, the authors state "In Drosophila, the DDC6/4-ET like complex (ME31B/Cup) is targeted for degradation in somatic cells". The Drosophila embryo has not yet cellularized at the time of these events, so "somatic region" should be used instead.

We have corrected this – thank you

Second decision letter

MS ID#: DEVELOP/2022/200920

MS TITLE: Specialized germline P bodies are required to specify germ cell fate in *C. elegans* embryos

AUTHORS: Madeline Cassani and Geraldine Seydoux
ARTICLE TYPE: Research Article

I am happy to tell you that your manuscript has been accepted for publication in Development, pending our standard ethics checks.

Reviewer 1

Advance summary and potential significance to field

Cassini and Seydoux make significant changes in the revised submission that add substantial clarity. This impressive study changes how we think about the dynamics and function of P granules and P bodies in primordial germ cells and their respective roles in PGC specification. Almost all of my previous points have been addressed or rectified. Images and quantifications in supplemental are thorough and complete. The additions that address the limitations of the study are appreciated.

Comments for the author

Previous critiques of figure 6 still remain in that there still appears to be more nos-2 and Y51F10.2 (and not less) in the meg-1/2 mutants. Granted, the increase is in the periphery, where P granules are likely silencing these transcripts. But because this isn't sufficiently clarified, most readers will still look at the meg-1/2 panel (especially with nos-2) and see more and not less.
I am guessing the method used to count nos-2 speckles discounts the bright P-granule localized blotches (or counts them as a single spot instead of many).
Spots detached from the nuclear periphery are clearly lower for all three transcripts in meg-1/2 mutants, and maybe that is what is being counted. But most readers will not pick up on that and wonder why the meg-1/2 images don't readily reflect the decreases quantified to the right.
A minor recommendation would be to add a short conclusion to line 294 reminding readers what the lower xnd-1 expression tells us about P4.

Reviewer 2

Advance summary and potential significance to field

See my review of the original submission.

© 2022. Published by The Company of Biologists under the terms of the Creative Commons Attribution License (https://creativecommons.org/licenses/by/4.0/).

Comments for the author

The authors' revisions and responses to reviewer comments address the issues that were raised and improve the manuscript. I do not have any further revisions to suggest.

Reviewer 3

Advance summary and potential significance to field

See previous review.

Comments for the author

I am satisfied with the revisions that the authors have made and support acceptance.

© 2022. Published by The Company of Biologists under the terms of the Creative Commons Attribution License (https://creativecommons.org/licenses/by/4.0/).
